# Use of buccal fat pad-derived stem cells cultured on bioceramics for repair of critical-sized mandibular defects in healthy and osteoporotic rats

**DOI:** 10.1007/s00784-022-04506-w

**Published:** 2022-05-07

**Authors:** Fabio Camacho-Alonso, M. R. Tudela-Mulero, J. A. Navarro, A. J. Buendía, A. M. Mercado-Díaz

**Affiliations:** 1 Department of Oral Surgery, University of Murcia, Murcia, Spain; 2 Oral Surgery Teaching Unit, University Dental Clinic, Morales Meseguer Hospital (2Nd Floor), Marqués de los Vélez s/n, 30008 Murcia, Spain; 3 Department of Histology and Pathological Anatomy, University of Murcia, Murcia, Spain; 4 Private Dental Practice, Murcia, Spain

**Keywords:** Bioceramics, Buccal fat pad mesenchymal stem cells, Mandibular symphysis, Bone regeneration, Osteoporosis

## Abstract

**Objective:**

To compare new bone formation in mandibular symphysis critical-sized bone defects (CSBDs) in healthy and osteoporotic rats filled with bioceramics (BCs) with or without buccal fat pad mesenchymal stem cells (BFPSCs).

**Materials and methods:**

Thirty-two adult female Sprague–Dawley rats were randomized to two groups (*n* = 16 per group): group 1 healthy and group 2 osteoporotic (with bilateral ovariectomy). The central portion of the rat mandibular symphysis was used as a physiological CSBD. In each group, eight defects were filled with BC (hydroxyapatite 60% and β-tricalcium phosphate 40%) alone and eight with BFPSCs cultured on BC. The animals were sacrificed at 4 and 8 weeks, and the mandibles were processed for micro-computed tomography to analyze radiological union and bone mineral density (BMD); histological analysis of the bone union; and immunohistochemical analysis, which included immunoreactivity of vascular endothelial growth factor (VEGF) and bone morphogenetic protein 2 (BMP-2).

**Results:**

In both groups, CSBDs filled with BC + BFPSCs showed greater radiological bone union, BMD and histological bone union, and more VEGF and BMP-2 positivity, compared with CSBDs treated with BC alone at 4 and 8 weeks.

**Conclusions:**

The application of BFPSCs cultured on BCs improves bone regeneration in CSBDs compared with BCs alone in healthy and osteoporotic rats.

**Clinical relevance:**

Our results may aid bone regeneration of maxillofacial CSBDs of both healthy and osteoporotic patients, but further studies are necessary.

**Supplementary Information:**

The online version contains supplementary material available at 10.1007/s00784-022-04506-w.

## Introduction

In recent decades, oral implantology has shown that oral rehabilitation of patients with single, multiple, or total dental losses is a predictable treatment with a high success rate in the short, medium, and long term [1]. However, the success of dental implants depends on the correct osseointegration of the implant, defined as the direct, structural, and functional connection between the living bone with remodeling capacity and the surface of the implant, without the interposition of fibrous tissue [2]. Maxillofacial critical-sized bone defects (CSBDs) can hinder implant rehabilitation in patients with large bone losses in the jaws due to trauma, osteonecrosis, ablative cancer surgery, or congenital deformities [3–6].

To reconstruct CSBDs, allografts, xenografts, and alloplastic biomaterials are usually ineffective, and conventional treatment is based on large volumes of bone from autografts (cortical, medullary, or corticomedullary), due to their biocompatibility and osteogenic properties [7]. Autografts may be of extraoral (skull, iliac crest, tibia, or rib) or intraoral (chin, anterior border of the mandibular ascending branch, maxillary tuberosity, and zygomatic flying buttress) origin [8]. However, autografts have disadvantages, such as the small amount of bone obtained (especially intraorally), morbidity, and intra- and postoperative complications at the donor site [9]. To avoid these problems, in recent years, new bone tissue engineering (BTE) techniques have been developed for the treatment of CSBDs, using various scaffolds on which mesenchymal stem cells (MSCs) are grown to create functional bone tissue that can be grafted in patients with large losses of maxillofacial bone [10–17].

MSCs are pluripotent stromal cells with a fibroblastoid morphology, which reside in a perivascular niche, and can differentiate into cells of mesodermal origin, such as neurons, myoblasts, adipocytes, chondrocytes, and osteoblasts [18]. The sources of MSCs include bone marrow, adipose tissue, placenta, pancreas, brain, trabecular bone, synovial membrane, peripheral blood, endometrium, hair follicle, umbilical cord, liver, dental pulp, and periodontal ligament [19]. To be considered MSCs, once obtained, they must meet the criteria proposed by the International Society for Cellular Therapy (SCT). First, they must be plastic adherent when maintained in standard culture conditions using tissue culture flasks. Second, ≥ 95% of the MSC population must express CD105, CD73, and CD90, as measured by flow cytometry. They must also lack expression (≤ 2% positive) of CD45, CD34, CD14 or CD11b, CD79α or CD19, and HLA-DR. Third, the cells must be able to differentiate to osteoblasts, adipocytes, and chondroblasts under standard in vitro differentiating conditions [20]. However, at present, concerning the second SCT criterion, cell characterization with four of the eight antigen markers is accepted to identify MSC: positivity of CD105, CD73, and CD90 and negativity of CD45 [21, 22]. Once the MSCs have been identified, different in vitro conditions permit using specific media supplementation to obtain the different cellular lineages [23].

The scaffolds used to seed these cells in BTE must be three-dimensional and porous, with a network of interconnected pores to allow nutrient transport. The chemical surface of the scaffold must allow for cell adhesion, proliferation, and differentiation, facilitating osteogenicity, osteoconductivity, and osteoinductivity for new bone formation [24]. Absorbable scaffolds used in BTE are divided into natural polymers, synthetic polymers, composite materials, and inorganic materials containing Ca/P [25, 26]. In recent years, inorganic materials have included bioceramics (BCs) composed of 60% hydroxyapatite Ca_10_(PO_4)6_OH_2_ (HA) and 40% of β tricalcium phosphate Ca_3_(PO_4) 2_ (β-TCP) with > 99 crystalline structure. In these BCs, the presence of HA delays the reabsorption of β-TCP, maintaining the three-dimensional volume of the scaffold, without affecting its properties [27]. BCs have been used in BTE as a scaffold for cultivating MSCs of various origins, such as human dental pulp cells [28], human jaw periosteal progenitor cells [29], bone marrow [30, 31], liver, synovial, muscle [32], and adipose tissue [33–35].

Traditionally, adipose stem cells (ASCs) have been obtained from visceral adipose tissue [36], orbital fat tissue [37, 38], and special fat pads such as the Hoffa pad [39], and the most frequently used donor-sites are the subcutaneous adipose tissue of the abdomen, breast, buttock, and thigh [16]. However, these procedures have shortcomings, such as harvested cells with heterogeneous populations [40], inadequate aspirate volume [41], donor site morbidity, painful interventions [42], and postoperative ambulatory difficulties [43]. To avoid these intra- and postoperative complications, the oral cavity contains a mass of specialized fatty tissue, named buccal fat pads (BFPs) or Bichat’s fat pads [44]. In the past four decades, numerous studies have used BFPs as an autogenous graft for the reconstruction of small- to medium-sized maxillofacial defects for closure of oroantral [45–50] and oronasal [45] communications, congenital cleft palate repair [51], reconstruction of the skull base and fronto-orbital defects [4], reconstruction of intraoral malignant defects [5, 52–54], zygomaticomaxillary reconstruction [6], palatoplasty [55], malar augmentation [56], osteonecrosis of the jaw [57], treatment of oral submucous fibrosis [58], treatment of temporomandibular joint ankyloses [59, 60], three-dimensional rehabilitation of large alveolar defects [17], closure of the perforation of the sinus membrane [61], treatment of severe gingival recessions [62, 63], pulp revascularization [64], and treatment of peri-implant mucosal defects [65]. In addition, they have recently been used to obtain MSCs called buccal fat pad mesenchymal stem cells (BFPSCs), because their features and behavior are similar to the better known subcutaneous mesenchymal stem cells (SCSCs) [16]. This new technique for obtaining BFPSCs has numerous advantages, since the harvesting of BFP is noncomplicated, requires minimal incision with local anesthesia, and causes minimal donor-site morbidity [66]. BFPSCs can be used in BTE for the regeneration of maxillofacial CSBDs from healthy patients, especially when systemic diseases, such as osteoporosis, affect the bone metabolism, resulting in reduced bone mass and, especially, density [35]. Proportionally increasing the number of mature osteoclasts and consequently increasing bone resorption means BTE could help regenerate oral CSBDs that can later be rehabilitated with dental implants.

Anatomically, the rat mandible consists of a pair of bones that never unite in adult life, as they do in other species; the two sides of the mandible are joined by fibrous tissue in the symphysis as a natural CSBD that has been used by three studies as a congenital nonunion model for investigating bone regeneration [31, 67, 68].

To date, the use in BTE of a construct of BC and BFPSCs for regeneration of CSBDs in healthy and osteoporotic subjects has not been studied. Thus, this study aimed to compare new bone formation in rat mandibular symphysis CSBDs using BC with or without BFPSCs in healthy and osteoporotic rats.

## Material and methods

Rats were supplied by the animal facility (REGA ES300305440012) (Research Support Unit) of the University of Murcia (Spain). The study protocol was approved by the University of Murcia Bioethics Committee (154/2015) and the competent local authority (A13180105) and followed the European Union Guidelines for animal experimentation (EU/63/2010). The research followed the requirements for the performance of Animal Research: Reporting of In Vivo Experiments (ARRIVE) guidelines and was conducted between May 2018 and February 2019. To calculate a representative sample size, a power of 80% was required (5% alpha level). Thirty-two adult female Sprague–Dawley (SD) rats were included in a prospective, randomized study.

The rats weighed between 247 and 255 g (mean 250 g) and were housed in individually ventilated cages with 12/12-h light/dark cycles, and food and water supplied ad libitum. The rats were provided with nutritionally balanced food (PB Panlab, Barcelona, Spain), crushed in advance (Robot Coupe®, Bourgogne, France) to provide a semi-soft diet, and were acclimatized for 1 week before study initiation.

### Randomization

Randomization was made using www.randomization.com. Rats were randomized to two groups (*n* = 16 per group): group 1 healthy and group 2 osteoporotic.

### Induction of osteoporosis

Osteoporosis was induced in group 2 (*n* = 16) by bilateral ovariectomy (OVX) [69]. Under aseptic conditions, the animals were anesthetized with a mixture of 50% ketamine (Ketamidor®, Richter Pharma AG, Wels, Austria) and 50% xylazine (Xilagesic®, Laboratories Calier S.A., Barcelona, Spain) administered by intraperitoneal injection at a dose of 0.1 mL/100 g body weight. When general anesthesia was achieved, a 10-mm linear incision was made in the lumbar lateral skin bilaterally. Locating the ovarian artery at a point between the lower margin of the free ribs and the iliac crest, an absorbable suture of 4/0 (Laboratories Normon S.A., Madrid, Spain) was placed around the ovarian artery and vein prior to the removal of both ovaries. The muscle layer was repositioned using 4/0 absorbable sutures (Laboratories Normon S.A., Madrid, Spain), and the skin was sutured using 4/0 nonabsorbable sutures (Ethilon®, Ethicon, Lidingö, Sweden). Analgesia was provided by subcutaneous administration of buprenorphine (Bupac®, Richter Pharma AG, Wels, Austria) 0.05 mg/kg before OVX. The success of bilateral OVX was assessed by analyzing the estrous cycle 2 weeks after surgery and the atrophy of the uterine horns after animal sacrifice [70]. The animals were not considered osteoporotic until 6 weeks after OVX [71].

### BFPSC isolation, culture, and osteogenic induction

**Fig. 1:**
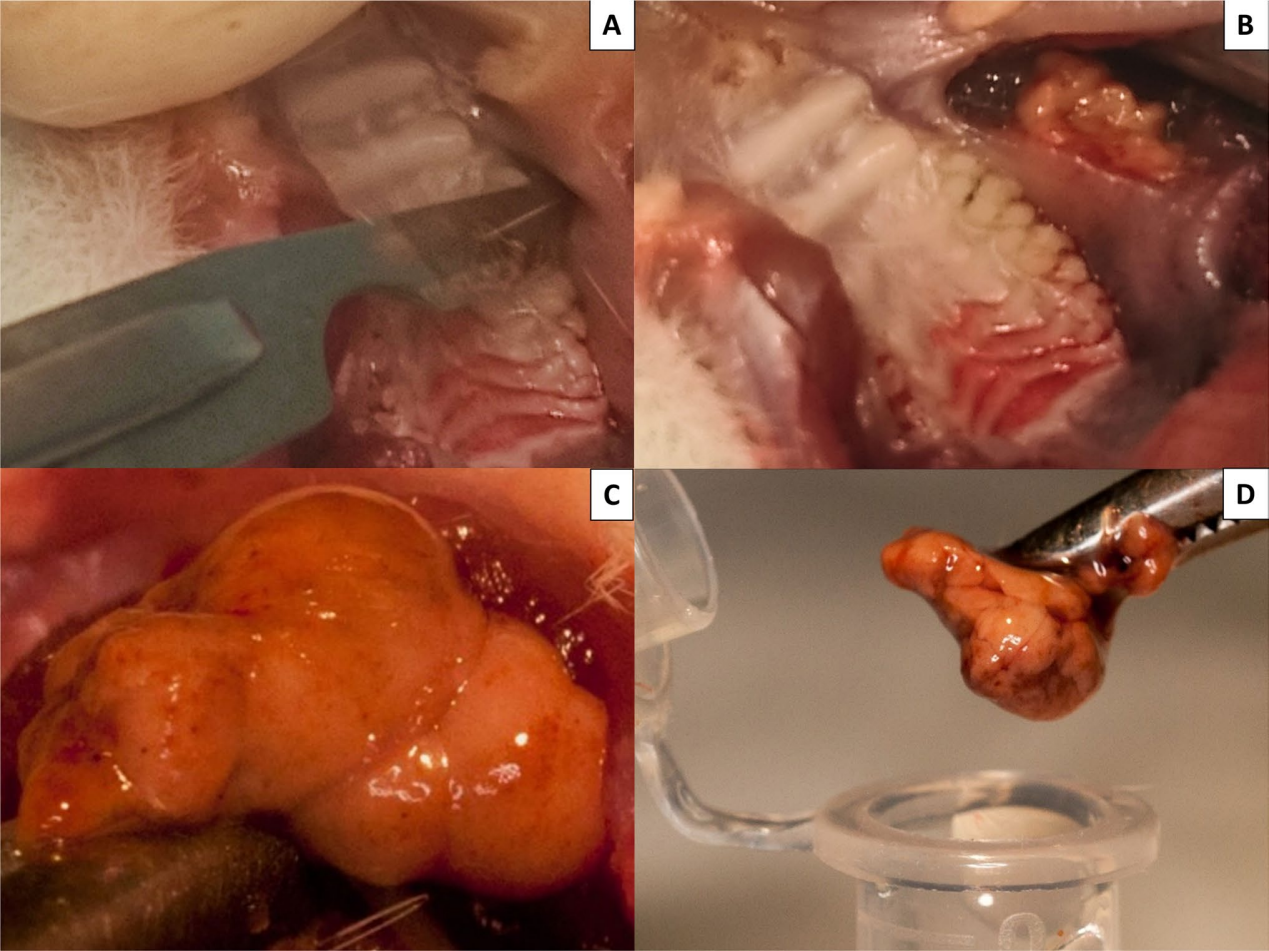
Removal of the left BFP. **A** Incision distal to the left maxillary second molar. **B** Location of the BFP. **C** Obtaining the BFP through gentle and careful dissection of soft tissues. **D** BFP introduced into PBS with antibiotic and antifungal

BCPSCs were obtained from two female Sprague–Dawley rats aged 4 weeks. After sacrifice, using CO_2_ inhalation under aseptic conditions, the four BFPs were accessed through a vestibular horizontal incision distal to the maxillary second molar (Fig. 1A). Once located (Fig. 1B), they were removed by gentle, careful soft tissue dissection (Fig. 1C), and were introduced into a sterile Eppendorf tube with 1.5 mL of sterile phosphate-buffered saline (PBS) to which antibiotic (1 mg/mL of streptomycin and penicillin) (Sigma-Aldrich Chemistry, S.A., Madrid, Spain), and antifungal (1 mg/mL of fungizone) (Sigma-Aldrich Chemistry, S.A., Madrid, Spain) (Fig. 1D) were added.

BFPSCs were obtained according to the technique described by Zuk et al. in 2002 [23] and Farré-Guash et al. in 2010 [66]. The four BFPs were minced into small pieces and treated with 0.075% collagenase I (Sigma-Aldrich Chemistry, S.A., Madrid, Spain) for 60 min at 37 °C. After incubation, adipose tissue was centrifuged at 400 g for 10 min to separate the adipocytes and lipid droplets from the stromal vascular fraction (SVF). Cell pellets were re-suspended in red blood cell lysis buffer (8.2 g/L NH_4_Cl, 0.84 g/L NaHCO_3_, and 0.37 g/L disodium ethylenediaminetetraacetic acid, pH 7.4) (Sigma-Aldrich Chemistry, S.A., Madrid, Spain) and incubated for 10 min at room temperature. The SVF was resuspended in low-glucose DMEM containing 10% fetal bovine serum (FBS) and 100 units/mL antibiotic/antimycotic solution (Sigma-Aldrich Chemistry, S.A., Madrid, Spain). Suspended cells were passed through a 100 μm cell strainer (BD, Biosciences, Palo Alto CA, USA), the cells were counted, and their viability was assessed with trypan blue exclusion. Cells were seeded at 5 × 10^3^ cells/cm^2^ in T-75 flasks in a humidified atmosphere of 95% air with 5% CO_2_ at 37 °C. After 3–4 days, individual colonies were visible on microscopic examination. The proliferated adherent cells were initially cultured to 80% confluence, harvested from the T-75 flasks using 0.05% trypsin (Sigma-Aldrich Chemistry, S.A., Madrid, Spain) and 0.53 mM ethylenediaminetetraacetic acid (EDTA) (Sigma-Aldrich Chemistry, S.A., Madrid, Spain) and resuspended at a cell density of 1 × 10^7^ cells/mL in culture medium. In this study, the adherent cells are referred to as BFPSCs.

BFP cells were induced to differentiate by an osteogenic medium containing DMEM, FBS 10%, 0.01 μm 1.25-dihydroxyvitamin D3, 50 μM ascorbic acid 2-phosphate, 10 mM β-glycerolphosphate, and 1% antibiotic/antimycotic solution (Sigma-Aldrich Chemistry, S.A., Madrid, Spain) for several weeks.

### Characterization of BFPSCs

To confirm the identity of isolated cells, the adherent isolated cells, considered BMSCs, were tested for the expression of membrane antigen markers (cluster of differentiation (CD)), using cells of passage 2: CD105 (Thermo Fisher, MA, USA), CD73 (BD Biosciences, San Diego, CA, USA), and CD90 (BD Biosciences, San Diego, CA, USA) markers, and negative expression markers CD45 (BD Biosciences, San Diego, CA, USA) hematopoietic markers using specific antihuman mouse monoclonal antibodies (CD105-AlexaFluor 488,CD73-APC,CD90-FITC,CD45-PE). Isotype controls for each fluorochrome (AlexaFluor 488, APC, FITC, and PE) were anti-mouse immunoglobulin (IgGa, k). Data were acquired with a BD LSRFortessa X-20 cytometer (Bio-Rad Laboratories Inc., Hercules, CA, USA).

### Determination of alkaline phosphatase activity

To confirm the success of osteogenic induction, we studied alkaline phosphatase (ALP) activity with an alkaline phosphatase detection kit (Sigma-Aldrich Chemistry, S.A., Madrid, Spain), using cells of passage 4 (density of 5 × 10^3^ cells/well) were seeded into 24-well plates and cultured in osteoinductive medium for 5 days according to the manufacturer’s instructions. Cells were washed twice with PBS and fixed with 4% paraformaldehyde diluted in PBS for 1–2 min at room temperature. After washing with TBST (tris-buffered saline with 0.05% Tween-20), the cells were exposed to a fast red violet solution (0.8 g/L) and Naphthol AS-BI phosphate solution (4 mg/mL) in AMPD buffer (2 mol/L) pH 9.5 and incubated at room temperature for 15 min. After this, the staining solution was aspirated and the wells were washed with TBST. Finally, the cells were covered with PBS and then were evaluated under an optical microscope Nikon Eclipse TE-2000U (Nikon Corp., Tokyo, Japan).

### BMSC culture on BC scaffold

Sterilized porous BCs (40 mg) composed of 60% HA and 40% β-TCP, with granulometry varying from 180 to 250 μm Osteosynt® (Eincobio Biomaterial Ltd., Belo Horizonte, MG, Brazil), were placed in one well of a 48-well polystyrene plate, and 1 mL cell suspension (using cells of passage 5) was added and cultivated for 48 h. Approximately 30% of viable cells adhered to the BCs, and therefore 7.5 × 10^5^ cells/10 mg BCs were used for the treatment of CSBDs. This process with 40 mg allowed the treatment of four animals (10 mg with BFPSCs per animal) and was therefore performed four times to treat the 16 animals whose CSBDs were filled with BCs + BFPSCs.

### Surgical procedure

**Fig. 2:**
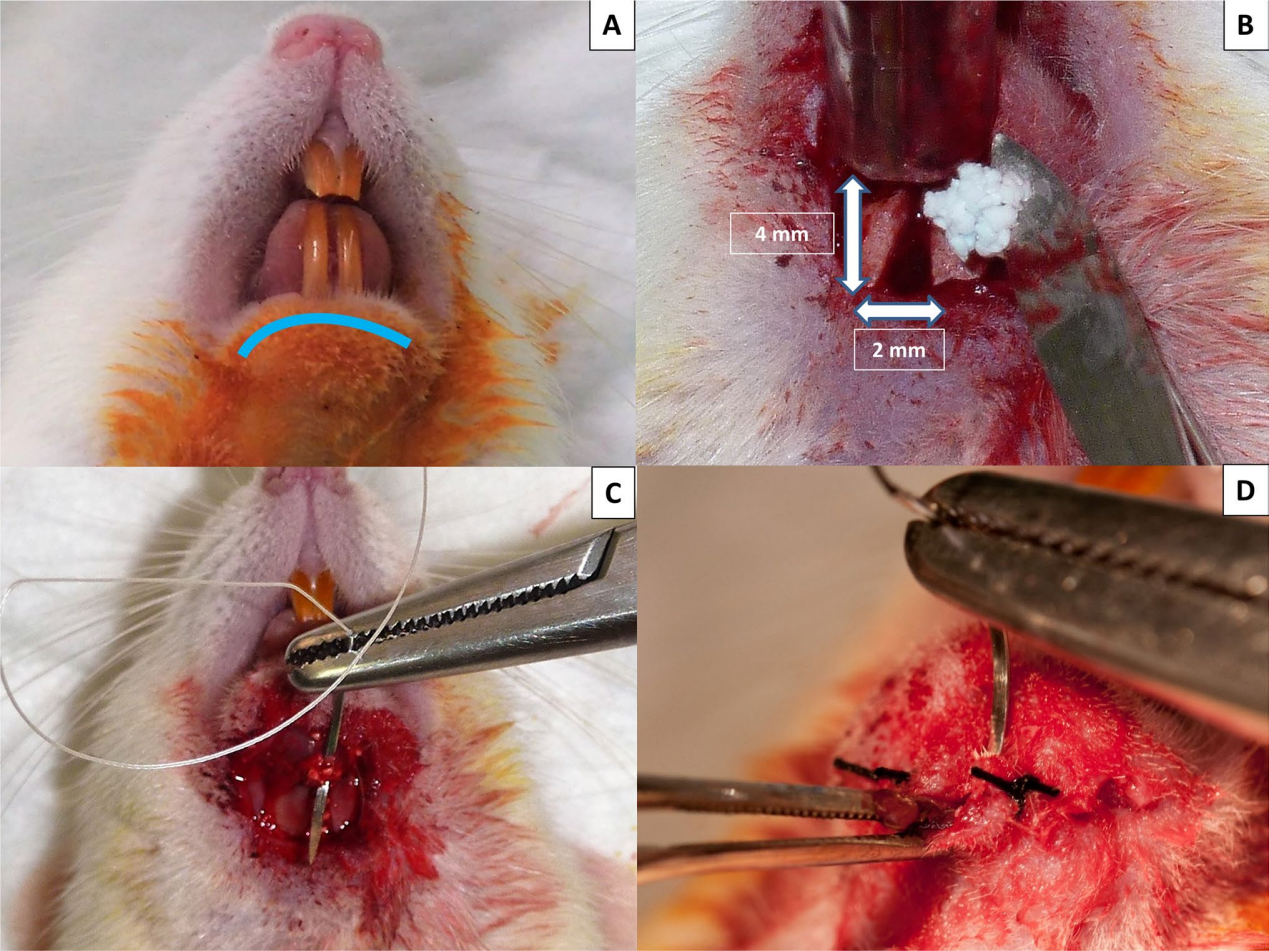
Creating and filling the CSBDs in the mandibular symphysis. **A** A 10-mm-long incision (crescent-shaped curving caudally) was made at the lower edge of the mandible. **B** Filling CSBDs (2 × 4 mm) in an osteoporotic animal with 10 mg porous BC cultured with 7.5 × 10^5^ BFPSCs with osteogenic induction. **C** Suturing the periosteum. **D** Suturing the skin

All animals were anesthetized with 50% ketamine (Ketamidor®, Richter Pharma AG, Wels, Austria) and 50% xylazine (Xilagesic®, Laboratories Calier S.A., Barcelona, Spain), administered by intraperitoneal injection at a dose of 0.1 mL/100 g body weight. When general anesthesia was achieved, local anesthesia of the mandibular symphysis was reinforced using articaine hydrochloride 4% with 1:100.000 epinephrine (Septodont, Saint-Maur-des-Fossés, France), achieving a local vasoconstrictor effect to reduce surgical bleeding. The submandibular region was shaved, washed with physiological serum, and covered with a 10% povidone-iodine solution. A 10-mm-long incision (crescent-shaped, curving caudally) was made at the lower edge of the mandible (Fig. 2A). The skin was elevated to expose the periosteum. An additional incision was made in the periosteum to expose the mandibular symphysis completely. Lastly, the fibrous tissues between the left and right mandibles were curetted, exposing the natural CSBD. The mean dimension of this defect in an adult 15-week-old female SD rat with an approximate weight of 250 g is 2 × 4 mm [24].

The CSBDs in the mandibular symphyses of eight animals in each group were filled with 10 mg porous BC Osteosynt^@^ (Eincobio Biomaterial Ltd., Belo Horizonte, MG, Brazil) (60% HA/40% β-TCP), while the CSBDs in the remaining eight animals were filled with 10 mg porous BC Osteosynt^@^ (Eincobio Biomaterial Ltd., Belo Horizonte, MG, Brazil) cultured with 7.5 × 10^5^ BFPSCs with osteogenic induction (Fig. 2B), according to the amount of BC and the number of BFPSCs proposed by Yagyuu et al. [31]. Planes were closed using 4/0 absorbable sutures (Laboratories Normon S.A., Madrid, Spain) to suture the periosteum (Fig. 2C) and the skin was sutured with braided silk (Lorca Marín S.A., Murcia, Spain) (Fig. 2D).

Analgesia for prospective pain control was provided by subcutaneous administration of 0.05 mg/kg buprenorphine (Bupac®, Richer Pharma AG, Wels, Austria) before surgery.

Half of the animals in each group (*n* = 8, four animals with CSBDs filled with BC, and four with defects filled with BC + BFPSCs) were euthanized in a chamber 4 weeks after surgery; the other eight animals in each group (four animals with CSBDs filled with BC, and four with defects filled with BC + BFPSCs) were euthanized 8 weeks after surgery. After sacrifice, the mandibles were processed by micro-computed tomography (micro-CT) to analyze radiological bone union, the histological analysis of bone union, and immunohistochemical analysis of immunoreactivity of VEGF and BMP-2.

### Radiological study (radiological bone union and bone mineral density)

The harvested rat mandibles were analyzed using a Skyscan 1172 micro-CT (Bruker®, Konitch, Belgium) and an Albira SPECT/PET/CT trimodal preclinical scanner (Bruker®, MA, USA).

For the Skyscan 1172 micro-CT (Bruker®, Konitch, Belgium), the following parameters were used: voltage of 50 kV, current 200 μA; samples were rotated 180°, and each mandible was scanned at intervals of 10 μm. An aluminum filter was used. NRecon software was used to reconstruct the X-ray projection images obtained during scanning. The sections were processed with CTAn and Data Viewer software (Bruker®, Konitch, Belgium). CTVox software (Bruker, Konitch, Belgium) was used to generate 3D models of the scanned samples.

The 3D images obtained by Skyscan 1172 micro-CT (Bruker®, Konitch, Belgium) were used for radiological examination of the mandibular symphysis union. The images were evaluated using the radiological scale proposed by Yagyuu et al. [31]: score 0 (no noticeable new bone formation), score 1 (cortical bone thickening along the margins of the mandibular symphysis), score 2 (bone union with apparent cracks/fissures), score 3 (bone union with or without a trace of cracks/fissures).

3D images obtained with the Albira SPECT/PET/CT trimodal preclinical scanner (Bruker®, MA, USA) were used for the bone mineral density (BMD) study. BMD was quantified at the midline of the mandibular symphysis in Hounsfield units (HU), using medical image data examiner software (AMIDE, UCLA, University, LA, USA). Four regions of interest (ROIs) were selected for each sample, with 1-mm^3^ volume boxes situated at the midline of the mandibular symphysis, calculating the mean of the four ROIs [68, 72].

### Histological bone union study

After micro-CT analysis, all mandibles (*n* = 32) were fixed on a 10% buffered formalin solution for 72 h, washed in distilled water for 5 min, and immersed in a decalcification solution of 10% (w/v) EDTA/PBS solution pH 7.4 at 25 °C. All specimens remained immersed in the decalcification solution for 10–12 days, gently agitating the samples the whole time and changing the EDTA every 3 days. Following paraffin embedding, 5-μm-tick coronal sections were cut. Hematoxylin and eosin (H&E) were used to reveal cellular detail and toluidine blue (TB) to stain mineralized bone. The histological sections were evaluated using the original scale reported by Salkeld et al. [73] and modified by Yagyuu et al. [31]: score 0 (fibrous union with a trace of new cartilage/bone formation), score 1 (fibrous union with some new cartilage/bone areas), score 2 (bone union with cartilaginous areas), and score 3 (complete bone union without cartilaginous areas).

### Immunochemical analysis

VEGF and BMP-2 were detected using two polyclonal antibodies developed in rabbits (ThermoFisher Scientific) and the avidin–biotin-peroxidase complex method (ABC). Briefly, after rehydration, the sections were washed in tris-buffered saline (TBS, 0.05 M, pH 7.6), treated with H_2_O_2_ 0.5% in methanol for 20 min, washed in TBS, and pretreated with normal swine serum (Dako, Carpinteria, CA, USA) diluted 1 in 100 in TBS to block nonspecific binding sites for 20 min. The sections were incubated with VEGF or BMP-2 antibody diluted 1 in 50 for 60 min, washed in TBS, and incubated for 20 min with a biotinylated antibody swine anti-rabbit IgG (Dako, Carpinteria, CA, USA), diluted 1 in 250. The slides were washed in TBS and incubated with ABC reagent (Vector Laboratories, Burlingame, CA, USA) for 20 min. Labeling was detected by incubation using the liquid DAB + substrate chromogen system (Dako, Carpinteria, CA, USA); the reaction was stopped after 5 min by rinsing the slides in tap water. Slides were counterstained in Mayer’s hematoxylin for 2 min, rinsed in tap water, and coverslipped. A positive reaction was identified by a dark-brown precipitate. There was no positive tissue immunoreaction when the primary antibodies were omitted or replaced by normal rabbit serum at the same concentrations as the primary antibody as negative controls. VEGF and BMP-2 expression were assessed by semiquantitative analysis using a scale of 0 to 3: score 0 (negative), score 1 (mild), score 2 (moderate), and score 3 (strong).

### Statistical analysis

Data were analyzed using the SPSS version 20.0 statistical package (SPSS® Inc., Chicago, IL, USA). A descriptive study was made of each variable. The Kolmogorov–Smirnov normality test and Levene’s homogeneity of variance test were applied, and the data showed a skewed distribution, so data were analyzed using a nonparametric ranking test. Semiquantitative (radiological bone union, histological bone union, and immunohistochemical analysis) and quantitative (BMD) data were evaluated using the Mann–Whitney *U*-test. Statistical significance was established as *p* ≤ 0.05.

## Results

All animals survived until the end of the study.

### BMSC characterization

**Fig. 3:**
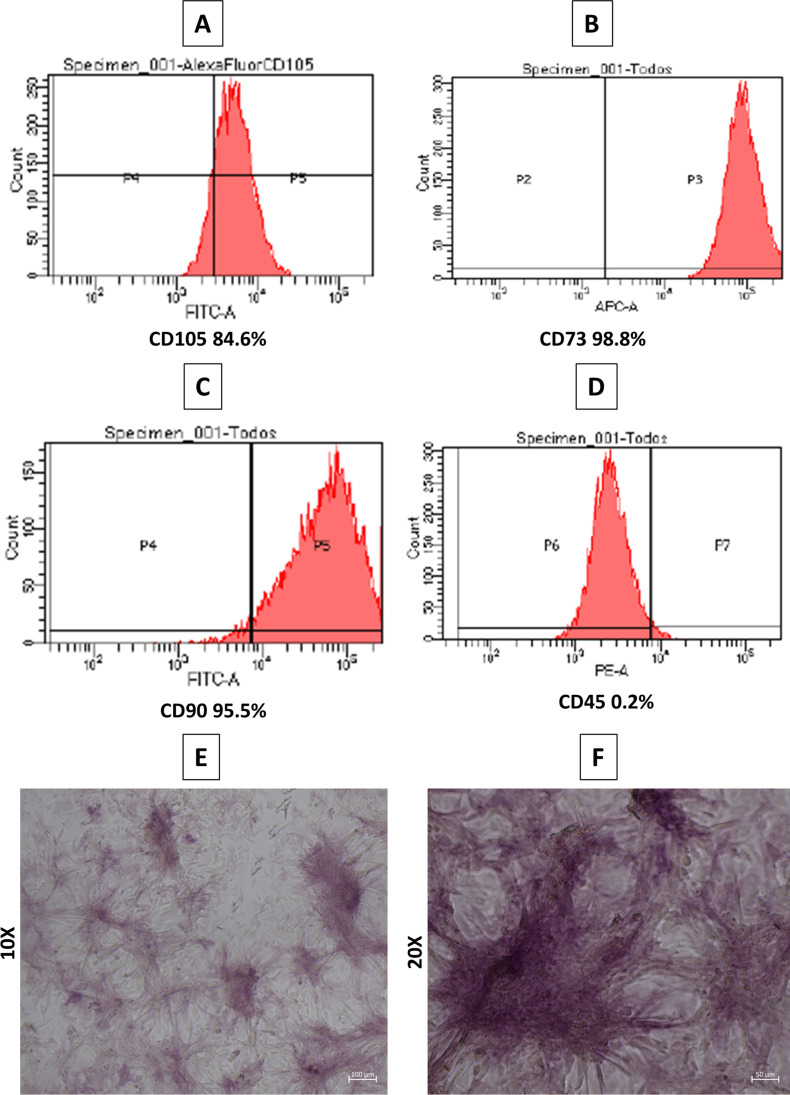
BMSC characterization and ALP activity. **A** Expression of CD105. **B** Expression of CD73. **C** Expression of CD90. **D** Expression of CD45. **E** and **F** ALP activity staining showed the presence of ALP-positive cells (10X and 20X)

The cells were positive for CD105 (84.6%) (Fig. 3A), CD73 (98.8%) (Fig. 3B), and CD90 (95.5%) (Fig. 3C) and negative for CD45 (0.2%) (Fig. 3D).

### ALP activity

The results of ALP activity are shown in Fig. 3E and F. After osteogenic induction, microscopic images of ALP activity staining clearly showed the presence of ALP-positive cells.

### Micro-CT analysis (radiological bone union and BMD)

**Fig. 4:**
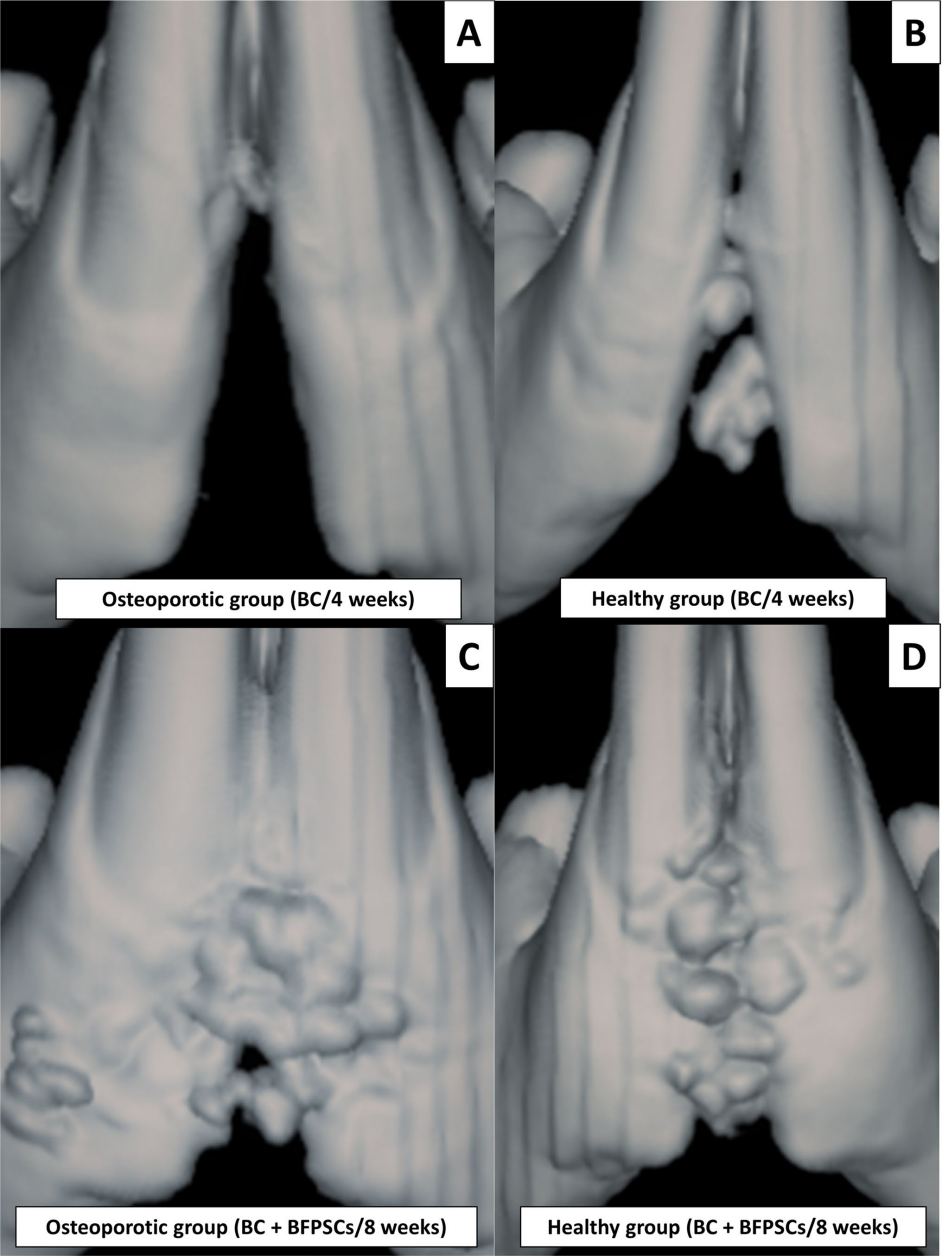
Micro CT analysis (radiological bone union). **A** Score 0 (no noticeable new bone formation), mandible at 4 weeks post-surgery of an osteoporotic rat treated with BC alone. **B** Score 1 (cortical bone thickening along the margins of the mandibular symphysis), mandible at 4 weeks after surgery of a healthy rat treated with BC alone. **C** Score 2 (bone union with apparent cracks/fissures), mandible at 8 weeks after surgery of an osteoporotic rat treated with BC + BFPSCs. **D** Score 3 (bone union with or without trace of cracks/fissures), mandible at 8 weeks after surgery of a healthy rat treated with BC + BFPSCs

In both study groups, the micro-CT radiological analysis indicated higher radiological bone union scores for BCs + BFPSCs filling than BCs alone with a statistically significant difference at both 4 weeks (group 1 *p* = 0.015 and group 2 *p* = 0.022) and 8 weeks after surgery (group 1 *p* = 0.008 and group 2 *p* = 0.018) (Table 1, Fig. 4). BMD was higher in both study groups when bone defects were filled with BCs + BFPSCs than when BCs were used alone, with statistically significant differences at both 4 weeks (group 1 *p* = 0.021 and group 2 *p* = 0.021) and 8 weeks after surgery (group 1 *p* = 0.020 and group 2 *p* = 0.021) (Table 2).

**Table 1:** Results of radiological union scale (Mann–Whitney *U*-test)

	Healthy		Osteoporotics	
Score	BCs (*n* = 4)	BCs + BFPSCs (*n* = 4)	BCs (*n* = 4)	BCs + BFPSCs (*n* = 4)
	*n* (%)	*n* (%)	*n* (%)	*n* (%)
Radiological union scale at 4 weeks				
0	1 (25)	0 (0)	3 (75)	0 (0)
1	3 (75)	0 (0)	1 (25)	1 (25)
2	0 (0)	3 (75)	0 (0)	3 (75)
3	0 (0)	1 (25)	0 (0)	0 (0)
Median (range)	1.00 (0.00–1.00)	2.00 (2.00–3.00)	0.00 (0.00–1.00)	2.00 (1.00–2.00)
*p*-value	0.015		0.022	
Radiological union scale at 8 weeks				
0	0 (0)	0 (0)	2 (50)	0 (0)
1	4 (100)	0 (0)	2 (50)	0 (0)
2	0 (0)	0 (0)	0 (0)	2 (50)
3	0 (0)	4 (100)	0 (0)	2 (50)
Median (range)	1.00 (1.00–1.00)	3.00 (3.00–3.00)	0.50 (0.00–1.00)	2.50 (2.00–3.00)
*p*-value	0.008		0.018

**Table 2:** Comparison between study groups of bone mineral density (BMD) in Hounsfield units (HU) (Mann–Whitney *U*-test)

Score	Healthy		Osteoporotics	
	BCs (*n* = 4)	BCs + BFPSCs (*n* = 4)	BCs (*n* = 4)	BCs + BFPSCs (*n* = 4)
BMD at 4 weeks				
Median (range)	261.34 (238.46–273.48)	450.33 (441.32–462.46)	101.51 (95.59–106.36)	219.10 (212.71–230.23)
*p*-value	0.021		0.021	
BMD at 8 weeks				
Median (range)	493.48 (476.29–502.35)	772.25 (757.41–790.16)	223.49 (209.79–225.29)	421.77 (412.41–428.19)
*p*-value	0.020		0.021	

### Histological analysis (histological bone union)

**Fig. 5:**
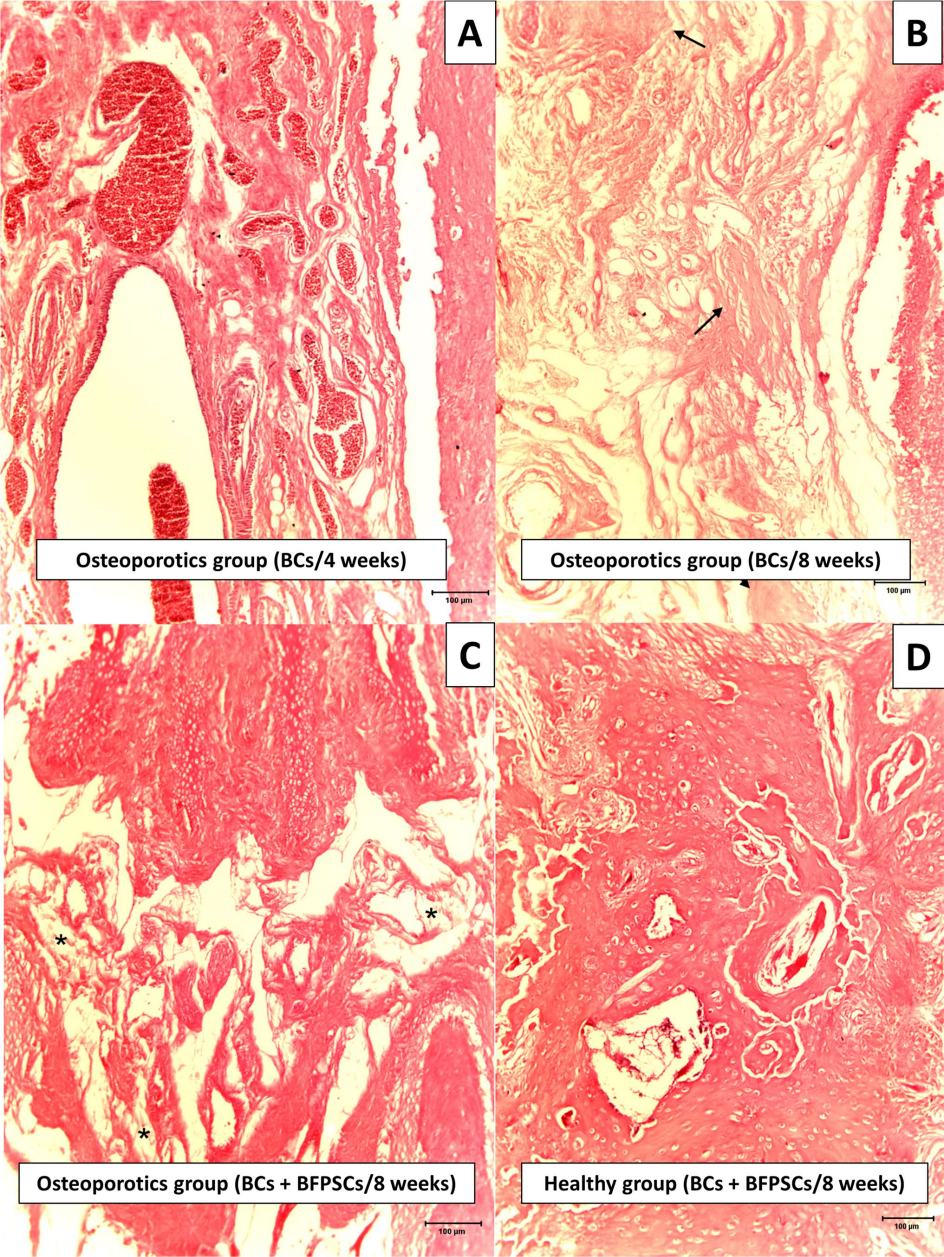
Histological bone union. **A** Score 0 (fibrous union with trace of new cartilage/bone formation), mandible at 4 weeks after surgery of an osteoporotic rat treated with BC alone. We observed fibrous union with blood vessels. **B** Score 1 (fibrous union with some new cartilage/bone areas), mandible at 8 weeks after surgery of an osteoporotic rat treated with BC alone. Fibrous union with small osteogenic areas (arrows). **C** Score 2 (bone union with cartilaginous areas), mandible at 8 weeks after surgery of an osteoporotic rat treated with BC + BFPSCs. Bone union with other fibrotic areas (asterisks). **D** Score 3 (complete bone union without cartilaginous areas), mandible at 8 weeks after surgery of a healthy rat treated with BC + BFPSCs

Histological analysis of bone union showed higher scores in both study groups when critical defects were filled with BCs + BFPSCs compared with BCs alone, with statistically significant differences at 4 weeks (group 1 *p* = 0.013 and group 2 *p* = 0.013) and 8 weeks after surgery (group 1 *p* = 0.011 and group 2 *p* = 0.017) (Table 3, Fig. 5).

**Table 3:** Results of histological union scale (Mann–Whitney *U*-test)

Score	Healthy		Osteoporotics	
	BCs (*n* = 4)	BCs + BFPSCs (*n* = 4)	BCs (*n* = 4)	BCs + BFPSCs (*n* = 4)
	*n* (%)	*n* (%)	*n* (%)	*n* (%)
Histological union scale at 4 weeks				
0	0 (0)	0 (0)	2 (50)	0 (0)
1	4 (100)	0 (0)	2 (50)	0 (25)
2	0 (0)	2 (50)	0 (0)	4 (100)
3	0 (0)	2 (50)	0 (0)	0 (0)
Median (range)	1.00 (1.00–1.00)	2.50 (2.00–3.00)	0.50 (0.00–1.00)	2.00 (2.00–2.00)
*p*-value	0.013		0.013	
Histological union scale at 8 weeks				
0	0 (0)	0 (0)	1 (25)	0 (0)
1	3 (75)	0 (0)	3 (75)	0 (0)
2	1 (25)	0 (0)	0 (0)	2 (50)
3	0 (0)	4 (100)	0 (0)	2 (50)
Median (range)	1.00 (1.00–2.00)	3.00 (3.00–3.00)	1.00 (0.00–1.00)	2.50 (2.00–3.00)
*p*-value	0.011		0.017

### Immunochemical analysis

**Fig. 6:**
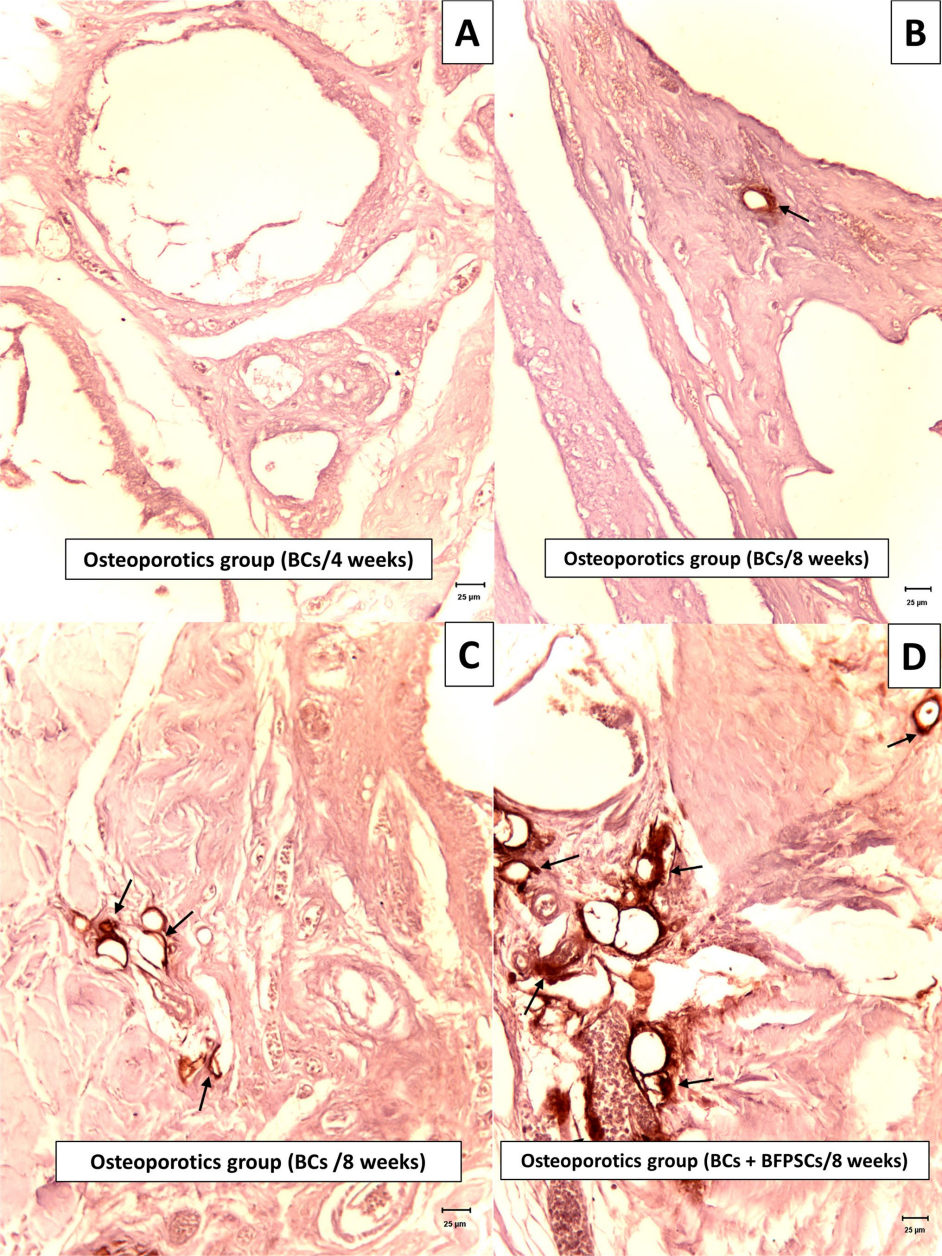
Expression of VEGF in mesenchymal cells (arrows). **A** Score 0 (negative), mandible at 4 weeks after surgery of an osteoporotic rat treated with BC alone. **B** Score 1 (mild), mandible at 8 weeks after surgery of an osteoporotic rat treated with BC alone. **C** Score 2 (moderate), mandible at 8 weeks after surgery of an osteoporotic rat treated with BC alone. **D** Score 3 (strong), mandible at 8 weeks after surgery of an osteoporotic rat treated with BC + BFPSCs

VEGF expression was observed in areas of ossification in poorly-differentiated mesenchymatous cells with fusiform or stellate morphology, ampullous cytoplasm, and euchromatic nuclei. In areas with well-differentiated bone tissue, labeling was observed in fusiform cells located in the periosteum and, to a lesser extent, in some osteoblasts. Occasionally, the vessels of the Havers ducts were marked. This analysis showed greater positivity in both study groups when critical defects were filled with BCs + BFPSCs compared with defects filled with BCs alone, with significant differences at 4 weeks in both groups (group 1 *p* = 0.040 and group 2 *p* = 0.013), and 8 weeks in group 2 (*p* = 0.013) (Table 4, Fig. 6).

**Table 4:** Results of VEGF expression (Mann–Whitney *U*-test)

Score	Healthy		Osteoporotics	
	BCs (*n* = 4)	BCs + BFPSCs (*n* = 4)	BCs (*n* = 4)	BCs + BFPSCs (*n* = 4)
	*n* (%)	*n* (%)	*n* (%)	*n* (%)
VEGF expression at 4 weeks				
0	0 (0)	0 (0)	2 (50)	0 (0)
1	0 (0)	0 (0)	2 (50)	0 (0)
2	4 (100)	1 (25)	0 (0)	4 (100)
3	0 (0)	3 (75)	0 (0)	0 (0)
Median (range)	2.00 (2.00–2.00)	2.00 (2.00–3.00)	0.50 (0.00–1.00)	2.00 (2.00–2.00)
*p*-value	0.040		0.013	
VEGF expression at 8 weeks				
0	0 (0)	0 (0)	1 (25)	0 (0)
1	0 (0)	0 (0)	2 (50)	0 (0)
2	1 (25)	0 (0)	1 (25)	0 (0)
3	3 (75)	4 (100)	0 (0)	4 (100)
Median (range)	3.00 (2.00–3.00)	3.00 (3.00–3.00)	1.00 (0.00–2.00)	3.00 (3.00–3.00)
p-*value*	0.317		0.013

**Fig. 7:**
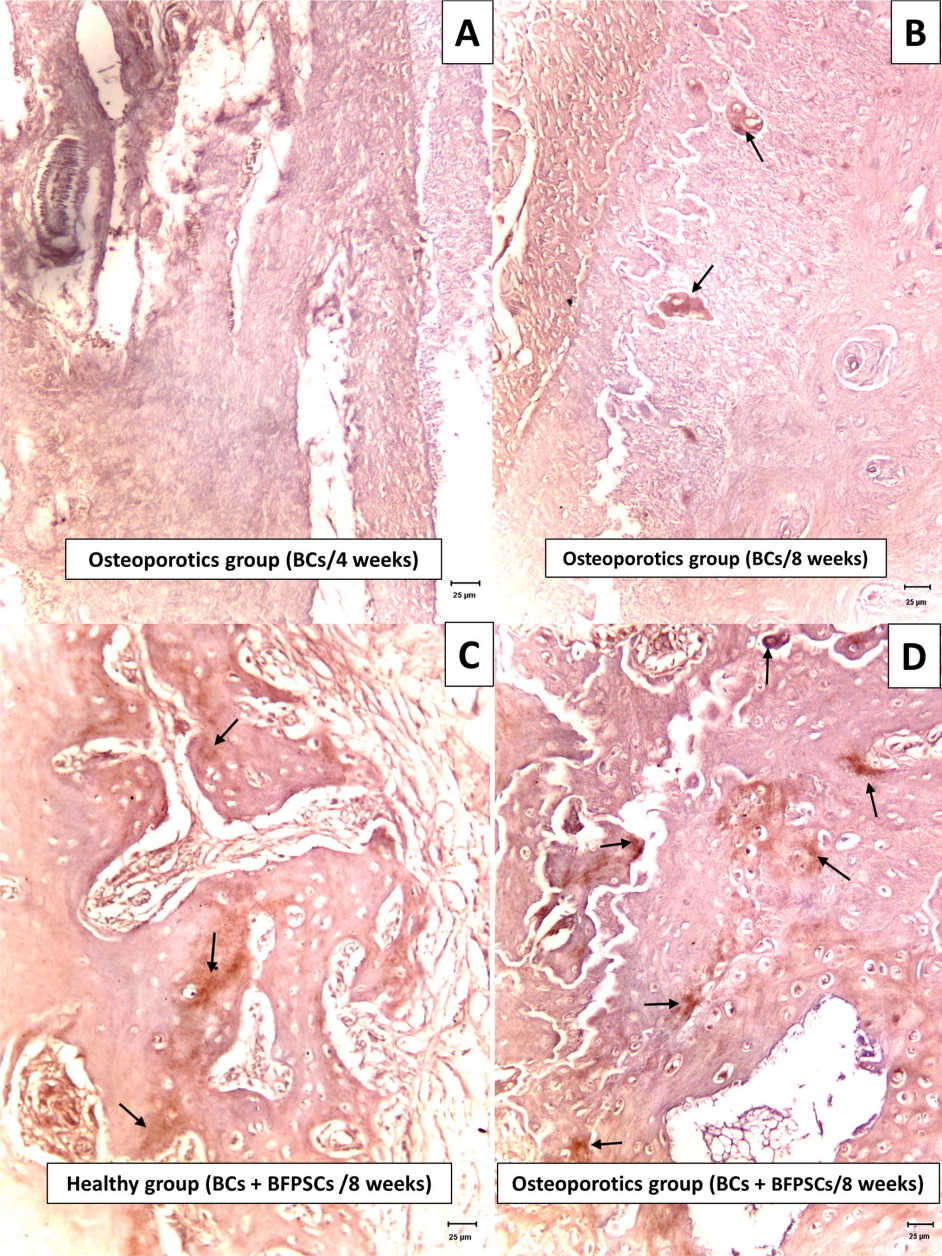
Expression of BMP-2 bone matrix surrounding osteoblastic cells (arrows). **A** Score 0 (negative), mandible at 4 weeks after surgery of an osteoporotic rat treated with BC alone. **B** Score 1 (mild), mandible at 8 weeks after surgery of an osteoporotic rat treated with BC alone. **C** Score 2 (moderate), mandible at 8 weeks after surgery of a healthy rat treated with BC + BFPSCs. **D** Score 3 (strong), mandible at 8 weeks after surgery of an osteoporotic rat treated with BC + BFPSCs

BMP-2 expression occurred in cells located in the bone matrix (osteocytes/osteoblasts) and was especially evident in the bone matrix surrounding these cells. This analysis showed greater positivity in the two groups when critical defects were filled with BCs + BFPSCs compared with defects filled with BCs alone, with significant differences in group 2, 8 weeks (*p* = 0.015) (Table 5) (Fig. 7).

**Table 5:** Results of BMP-2 expression (Mann–Whitney *U*-test)

Score	Healthy		Osteoporotics	
	BCs (*n* = 4)	BCs + BFPSCs (*n* = 4)	BCs (*n* = 4)	BCs + BFPSCs (*n* = 4)
	*n* (%)	*n* (%)	*n* (%)	*n* (%)
BMP-2 expression at 4 weeks				
0	0 (0)	0 (0)	2 (50)	0 (0)
1	2 (50)	0 (0)	2 (50)	2 (50)
2	2 (50)	4 (100)	0 (0)	2 (50)
3	0 (0)	0 (0)	0 (0)	0 (0)
Median (range)	1.50 (1.00–2.00)	2.00 (2.00–2.00)	0.50 (0.00–1.00)	1.50 (1.00–2.00)
*p*-value	0.127		0.061	
BMP-2 expression at 8 weeks				
0	0 (0)	0 (0)	1 (25)	0 (0)
1	0 (0)	0 (0)	3 (75)	0 (0)
2	4 (100)	2 (50)	0 (0)	3 (75)
3	0 (0)	2 (50)	0 (0)	1 (25)
Median (range)	2.00 (2.00–2.00)	2.50 (2.00–3.00)	1.00 (0.00–1.00)	2.00 (2.00–3.00)
*p*-value	0.127		0.015

## Discussion

In our study (in both healthy and osteoporotic rats), CSBDs filled with BC + BFPSCs showed greater radiological bone union, BMD and histological bone union, and more VEGF and BMP-2 positivity, compared with CSBDs treated with BC alone at 4 and 8 weeks.

In recent years, to avoid the main disadvantages of bone autografts (difficulty in obtaining large amounts of bone, morbidity, and complications at the donor site), in addition to the use of allografts, xenografts, and different alloplastic biomaterials, other reconstructive techniques such as bone distraction and BTE have been described. Osteogenic distraction is based on the progressive elongation of bone fragments, and although it has some advantages concerning autologous bone grafts (does not require a second surgical field to acquire the graft and a simultaneous elongation of the adjacent soft tissues), its disadvantages include the need for a distractor adapted to the CSBDs, a high risk of infection, and the difficulty of controlling the growth of the bone segments [74, 75].

BTE consists of the use of three-dimensional scaffolds on which osteoblastic stem cells or bone progenitors are grown [10, 11]. The choice of scaffold is a fundamental element in BTE for successful, rapid bone regeneration. An ideal scaffold should be three-dimensional and highly porous, with a network of interconnected pores to allow cell growth and the transport of nutrients and metabolic waste. Although natural and synthetic polymers are bioabsorbable, their major drawback is that the by-products of their degradation might provoke an undesirable reaction in the body [24], for this reason, scaffolds based on inorganic materials containing Ca/P are more frequently used, especially those composed of 60% HA and 40% β-TCP since, in these BCs, HA delays the reabsorption of β-TCP, favoring slower reabsorption of the scaffold by coinciding with tissue growth [27].

The next step after selecting the right scaffold is to choose a reliable source of cells that allows for their isolation and spread. MSCs are used as a viable alternative for the regeneration of numerous tissues. MSCs are pluripotential, and can, under certain conditions, differentiate into cells lineages including bone, nerve structures, cartilage, fat, skin, tendon, and muscle [76]. The most common sources of MSCs are bone marrow and adipose tissue. The many advantages of adipose tissue as a source of stem cells include large amounts are available, they are easy to obtain with limited morbidity, the cellular performance is much higher than that found in other sources, the cells are easily isolated from the other cellular components of the tissue, and the in vitro expansion procedure is simple, so they have a higher proliferation rate than bone marrow stromal stem cells (BMSCs) [77–79]. In addition, ASCs have a lower risk of rejection and are more genetically stable in long-term cultures, with a lower senescence rate than BMSCs [80, 81]. Likewise, they secrete VEGF and anti-inflammatory proteins against inflammatory molecules and play an important role in angiogenesis and bone repair [79]. Thus, BFP is a rich source of ASCs and might play an important future role in maxillofacial CSBD treatments using BTE techniques with a construct of BC and BFPSCs, which requires minimal intraoral incision with local anesthesia (minimal donor-site morbidity) [66].

Our methodology avoided the action of natural regenerative processes after creating an experimental iatrogenic CSBD, as these could alter the analysis of the biomaterial investigated. We did not create a bone defect but took advantage of the fact that the rat mandible does not present continuous bone in the mandibular symphysis; instead, it has fibrous connective tissue that interposes between the left and right sides of the mandible. This particularity makes the rat mandibular symphysis a naturally occurring bone defect that has been used by various researchers as a congenital nonunion model in bone regeneration trials. In 2015, Yagyuu et al. [31] used this natural CSBD for the assessment of cell therapy, comparing two types of treatment for bone regeneration of the CSBD: β-TCP alone or β-TCP with BMSCs. In 2016, Ueyama et al. [67] used the same model to compare the regenerative capacity of osteogenic matrix cell sheets with untreated defects. In 2020, Camacho-Alonso et al. [68] used this congenital nonunion model to compare new bone formation in CSBDs rats filled with HA alone and HA combined with simvastatin.

Our results show that in both study groups, CSBDs filled with BC + BFPSCs presented greater radiological bone union, BMD, histological bone union, and more VEGF and BMP-2 positivity, in comparison with CSBDs treated with BC alone (at 4 and 8 weeks). To date, the use in BTE of a construct of BC and BFPSCs for regeneration of CSBDs in healthy and osteoporotic subjects remains unclear. Obtaining BFPSCs from BFP with characteristics similar to ASCs from subcutaneous adipose tissue (SC-ASCs) has been demonstrated in several in vitro studies in animal [82] and human cells [16, 66]. In 2013, Niada et al. [82] isolated ASCs from interscapular subcutaneous adipose tissue and the BFP of six swine. Cells were characterized for their stemness and multipotent features. Their osteogenic ability when cultured on titanium disks and silicon carbide-plasma-enhanced chemical vapor-deposition fragments and their growth in the presence of autologous and heterologous serum were also assessed. The authors concluded that swine BFP contains progenitor cells with mesenchymal features and osteo-differentiate well in association with synthetic supports and suggested that porcine BFPSCs may be applied for maxillofacial bone-defect regeneration. In the same year, Broccaioli et al. [16] compared the features of BFPSCs with human SC-ASCs. They showed an important clonogenic ability and the typical mesenchymal stem cell immunophenotype. When correctly induced, osteogenic and adipogenic differentiation markers, such as alkaline phosphatase activity, collagen deposition, and lipid vacuole formation, were observed. Growths of both BFPSCs and SC-ASCs in the presence of human serum and their adhesion to natural and synthetic scaffolds were also assessed. Both types of ASCs adapted rapidly to human autologous or heterologous sera, increasing their proliferation rate compared with standard cultures, and all cells adhered finely to bone, periodontal ligament, collagen membrane, and polyglycol acid filaments (present in the oral cavity and commonly used in oral surgery). The authors concluded that BFP contains BFPSCs with stemness features that can differentiate and adhere to biological supports and synthetic materials. Therefore, the authors proposed BFPSCs for maxillofacial BTE. Similarly, Farré-Guasch et al. [66] analyzed the stromal vascular fraction obtained from fresh human BFP-derived adipose tissue to detect and quantify the percentage of ASCs in this tissue. The results showed that BFP contains a population of SC that share a similar phenotype with SC-ASCs and can also differentiate into the chondrogenic, adipogenic, and osteogenic linage. Therefore, the authors defined BFP as a new, rich, and accessible source of ASCs for maxillofacial BTE.

Concerning the good in vivo results we obtained using a BC composed of 60% HA/40% β-TCP as a scaffold for the culture of BFPSCs, two recent in vitro studies investigated the proliferation and osteogenic differentiation of BFPSCs grown in HA without other drugs [12] or combined with drugs [83]. In 2019, Hosseini et al. [12] compared the osteogenic differentiation potential by growing three human SCs on a scaffold of HA-coated electrospun polycaprolactone (PCL): BFPSCs, BMSCs, and unrestricted somatic stem cells (USSCs). SCs proliferation and osteogenic differentiation were investigated using 3-(4,5-dimethyl-2-thiazolyl)-2,5-diphenyl-2H-tetrazolium bromide (MTT assay), alizarin red staining, alkaline phosphatase activity, calcium content, and gene expression assays. Due to the availability, facilitated preparation procedure, and lower morbidity, the authors stated that BFPSCs are a better choice than BMSCs and USSCs for use in BTE. In 2020, Mahdavi et al. [83] manufactured a novel gelatin (G)-HA-/vitamin D (VD)–loaded graphene oxide (GO) scaffold, on which they grew BFPSCs. MTT assay, alkaline phosphatase activity, and cell adhesion were used to evaluate in vitro biological responses. The results demonstrated correct cellular growth and adequate osteogenic differentiation of the BFPSCs on this new scaffold.

Although few clinical studies in humans have used BFPSCs for bone regeneration of maxillofacial bone defects, in 2018, Khojasteh et al. [11] performed an exploratory prospective clinical study that evaluated and compared the efficacy of regenerating posterior mandibular atrophy with BFPSCs combined with inorganic bovine bone mineral at 50% or by particulated autologous bone in 14 patients. They concluded that BTE using BFPSCs may provide an alternative to autogenous bone reconstruction of alveolar ridge defects. In 2019, Akhalaghi et al. [84] studied nine patients with bone defects, who were allocated to two study groups: iliac crest bone graft with human amniotic membrane (HAM) coverage (*n* = 5) and iliac bone graft covered with HAM loaded with BFPSCs (*n* = 4). Five months after the graft and prior to implant placement, cone beam computed tomography was performed for radio morphometric analysis. The authors concluded that HAM with BFPSCs may enhance bone regeneration, especially in the horizontal dimension.

We found no studies with which to compare our results. However, Lin et al. [85] performed a total of 12 calvarial 5 mm CSBDs in six Fisher 344 female osteoporotic rats. CSBDs were randomized to two treatment groups: porous Sr-substituted calcium silicate (SrCs) ceramic alone or SrCs ceramic + BMSCs. At 4 weeks, the newly-formed bone area in CSBDs treated with SrCs ceramic + BMSCs was greater than that treated with SrCs ceramic alone, and the BMD was significantly higher. These, and our results, suggest BFPSCs may be used in BTE for the regeneration of maxillofacial CSBDs in healthy patients, but especially in osteoporotic patients who have lost bone mass and density, and where the regeneration of three-dimensional bone defects in the maxillofacial region may be difficult [35].

One of the main limitations of the study was that we could not compare the results with other studies, as this is the first study to compare new bone formation in rat mandibular symphysis CSBDs using BCs with or without BFPSCs in healthy and osteoporotic rats. In addition, another limitation of this study is that only the authors [68] and few researchers [31, 67] have used the rat mandibular symphysis as a model for bone regeneration, perhaps the results of this study could be different when the technique proposed will be applied in large bone defects due to trauma, osteonecrosis, or ablative cancer surgery.

In conclusion, the application of BFPSCs cultured on BCs improves bone regeneration in CSBDs compared with the application of BCs alone in healthy and osteoporotic rats.

## Supplementary Information

Below is the link to the electronic supplementary material.

Supplementary file1 (PDF 2488 kb)
